# Malaria control in Bhutan: case study of a country embarking on elimination

**DOI:** 10.1186/1475-2875-11-9

**Published:** 2012-01-09

**Authors:** Thinley Yangzom, Cara Smith Gueye, Rinzin Namgay, Gawrie NL Galappaththy, Krongthong Thimasarn, Roly Gosling, Shiva Murugasampillay, Vas Dev

**Affiliations:** 1 Vector-Borne Disease Control Programme, Ministry of Health, Royal Government of Bhutan, Post Box 191, Gelephu, Bhutan; 2 Global Health Group, University of California, San Francisco, 50 Beale Street, Suite 1200, San Francisco, CA, USA; 3 National Malaria Control Programme, Ministry of Health, Public Health Complex, 555/5, Elvitigala Mawatha, Colombo 05, Sri Lanka; 4 Malaria Unit, South-East Asia Regional Office of the World Health Organization, World Health House, Indraprastha Estate, Mahatma Gandhi Marg, New Delhi 110 002, India; 5 Global Malaria Programme, World Health Organization, Avenue Appia 20, 1211, Geneva 27, Switzerland; 6 National Institute of Malaria Research (ICMR), Chachal, VIP Road, Guwahati 781 022, Assam, India

**Keywords:** Bhutan, Malaria, Elimination, Control, Migration, Migrant, Diagnosis, Treatment, Surveillance, Vector

## Abstract

**Background:**

Bhutan has achieved a major reduction in malaria incidence amid multiple challenges. This case study seeks to characterize the Bhutan malaria control programme over the last 10 years.

**Methods:**

A review of the malaria epidemiology, control strategies, and elimination strategies employed in Bhutan was carried out through a literature review of peer-reviewed and grey national and international literature with the addition of reviewing the surveillance and vector control records of the Bhutan Vector-Borne Disease Control Programme (VDCP). Data triangulation was used to identify trends in epidemiology and key strategies and interventions through analysis of the VDCP surveillance and programme records and the literature review. Enabling and challenging factors were identified through analysis of socio-economic and health indicators, corroborated through a review of national and international reports and peer-review articles.

**Findings:**

Confirmed malaria cases in Bhutan declined by 98.7% from 1994 to 2010. The majority of indigenous cases were due to *Plasmodium vivax *(59.9%) and adult males are most at-risk of malaria. Imported cases, or those in foreign nationals, varied over the years, reaching 21.8% of all confirmed cases in 2006.

Strategies implemented by the VDCP are likely to be related to the decline in cases over the last 10 years. Access to malaria diagnosis in treatment was expanded throughout the country and evidence-based case management, including the introduction of artemisinin-based combination therapy (ACT) for *P. falciparum*, increasing coverage of high risk areas with Indoor Residual Spraying, insecticide-treated bed nets, and long-lasting insecticidal nets are likely to have contributed to the decline alongside enabling factors such as economic development and increasing access to health services.

**Conclusion:**

Bhutan has made significant strides towards elimination and has adopted a goal of national elimination. A major challenge in the future will be prevention and management of imported malaria infections from neighbouring Indian states. Bhutan plans to implement screening at border points to prevent importation of malaria and to targeted prevention and surveillance efforts towards at-risk Bhutanese and migrant workers in construction sites.

## Background

In recent years, there has been substantial progress made in reducing the malaria burden around the globe [1,2]. From the deep Amazon and the coastal plains of East Africa to the Malaysian peninsula, incidence has been decreasing over the last decade, related to increased resources for malaria control and better access to new and improved tools [3,4]. The South-East Asia region has some of the most pronounced declines, with five countries out of 11 reporting decreases of more than 50% of cases from 2000 to 2009 [2]. One of these success stories is tucked away, high up in the eastern Himalayas: Bhutan has achieved remarkable success in bringing down malaria transmission and announced a national strategy to eliminate malaria by 2016.

The progress made in Bhutan in the last 10 years is remarkable given the major challenges it faces. The country is placed in some of the most difficult terrain in the region, where landslides create impassible roads in the monsoon months and where 21% of households are located more than 4 h walk from the nearest road [5]. The low-lying southern region of Bhutan is at high-risk for malaria transmission [6] and has a highly porous border with India, through which there is significant population movement. In addition, large numbers of migrant workers enter the country to work in large-scale development projects in areas vulnerable to malaria transmission, creating a risk of continual importation and re-introduction of malaria into the area [7]. Given these and other challenges, the recent success in reducing malaria incidence may contain lessons for other countries [8].

This paper seeks to characterize the malaria programme of Bhutan over the last 10 years, exploring trends in the epidemiology of malaria, malaria control strategies and interventions, and the enabling and challenging conditions of Bhutan with emphasis on the endemic southern border and population migration.

## Methods

### Geography, population and climate

The Royal Government of Bhutan is a small country, spanning 38,394 km^2^, with a population of 677,343 bordered in the north by the Tibetan Region of China, and by India to the west, south and east with the states of Sikkim, West Bengal, Assam, and Arunachal Pradesh, respectively. The country is mainly mountainous, rising to a maximum elevation of 7,314 m and extending down to as low as 160 m above mean sea level in the southern foothills. Bhutan's economy is based on agriculture, forestry, and hydropower electricity exports to India [9,10].

Bhutan has seasonal rainfall with monsoon rains occurring from June to September, when most malaria cases occur [11]. A winter northeast monsoon occurs from November to March, with snowfall in the higher elevations. Bhutan has only 2.3% arable land, most of which is in the west [10].

**Figure 1:**
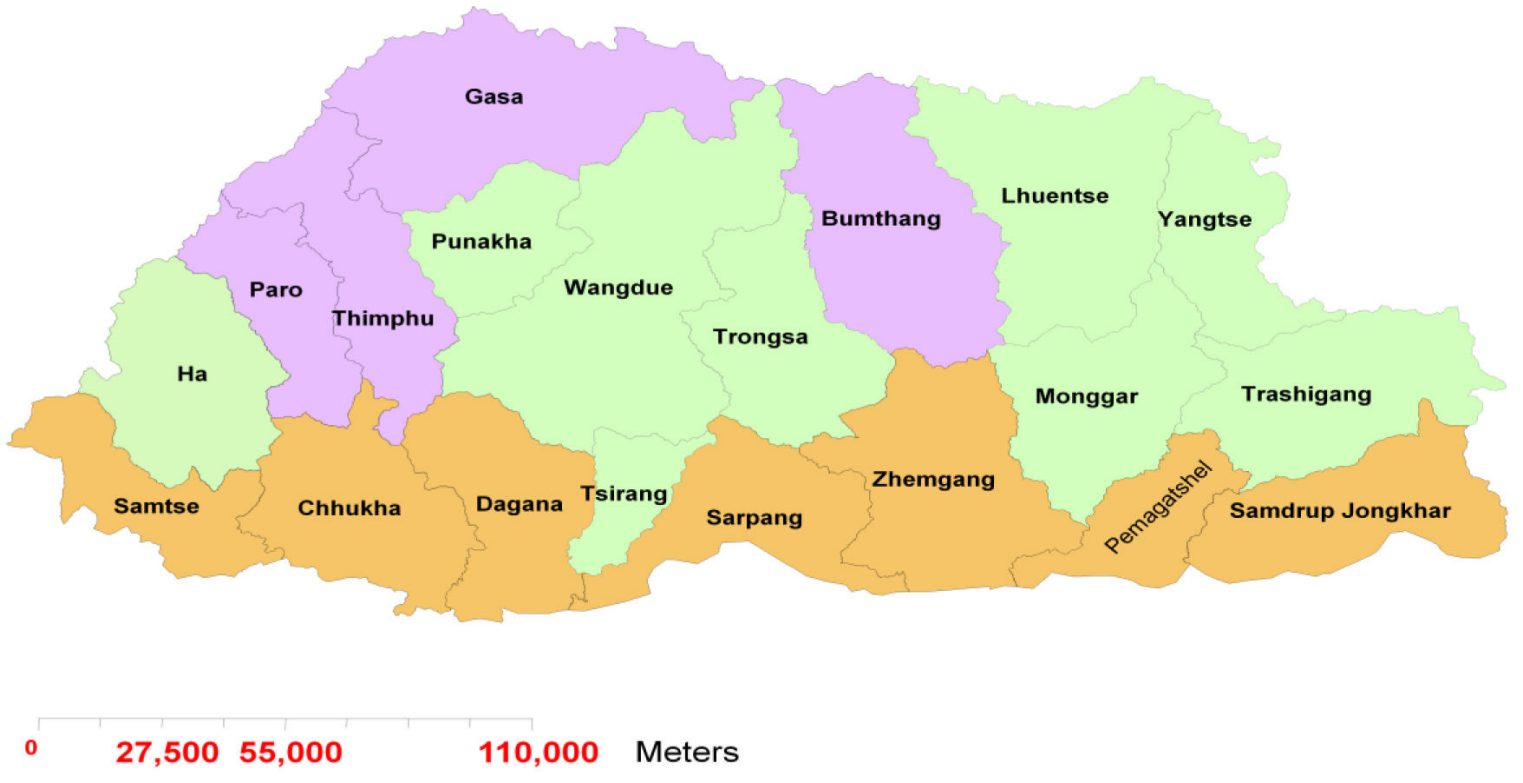
**Prevalence of malaria in Bhutan**. Districts in purple are malaria-free, districts in green are considered at risk for seasonal transmission, districts in brown are considered malaria-endemic.

Malaria risk areas are mainly forest and forest-fringe human settlements, in particular those with irrigation or development projects, such as hydropower project sites [5]. Twenty-four percent of the population lives in areas considered free of malaria, located in four districts in the north-east and central part of the country (Figure 1) [12]. These areas are not receptive to malaria transmission due to their high elevation and cooler temperatures. Indigenous cases reported from these districts are imported cases from other districts. Nine districts in a band running east to west across the center of the country are considered at risk for seasonal transmission, having a history of local transmission although some of them have not had an indigenous case in the last 3 years. This zone contains 34% of the population. Seven districts, with a population of 284,512 (42% of the total population), are considered malaria-endemic, where transmission occurs throughout the year [11]. These districts border the Indian states of Assam and West Bengal.

### Programme data

A literature review was conducted using PubMed, Google Scholar, Google, SpringerLink, [13] World Health Organization (WHO) South-East Asia Region Institutional Repository, [14] World Health Organization Library Database (WHOLIS), [15] and through requests to the WHO Archives at the WHO Headquarters in Switzerland. Search terms were "Bhutan" AND "malaria" OR "prevention" OR "refugee" OR "Nepal" OR "India" OR "supply, supply system" OR "health system" OR "health supply."

Routine national health facility surveillance data were collected and reviewed in-country by two researchers (TY and CSG), for indigenous (cases contracted locally) and imported cases [16]. In Bhutan, an imported case is a confirmed malaria infection in any foreign national. The reported cases described in this study were confirmed by microscopy. Reporting of presumptive cases is not the policy in Bhutan and rapid diagnostic test (RDT) results are cross checked by microscopy. Results of the microscopy confirmation process are reported, not the RDT results. These secondary data were collected from the Vector-borne Disease Control Programme (VDCP) headquarters in Gelephu, which receives and compiles reports of confirmed cases from the districts. Other data collected were VDCP-derived estimates of population at risk and distribution and coverage of long-lasting insecticidal net (LLIN), insecticide-treated bed nets (ITN), and Indoor Residual Spray (IRS). Annual rainfall data were collected by the VDCP for the period 1996-2010 for 18 districts. When discrepancies between any of the data sources were found, follow-up information was sought from district offices by the VDCP programme manager (TY).

### Data analysis

Surveillance and vector control data were plotted using Microsoft Excel and trends were observed. These trends were then compared with those described in the literature identified in the review and with information provided by the VDCP headquarters and district officers, using data triangulation to identify and confirm trends [17]. The WHO World Malaria Report surveillance data were used to corroborate the VDCP data records.

### Ethical considerations

The Ministry of Health in Bhutan approved the conduct of the case study. Data from the Ministry of Health, Vector-Borne Disease Control Programme were analysed in aggregate form.

## Findings

### Literature review

The literature review identified 35 peer-review publications, 28 WHO reports and documents, ten reports by other partners and agencies, and 20 published or grey reports from Bhutanese ministries including three reports from the Ministry of Health. A list of these documents is shown in the online web appendix (Additional file 1) [18]. These documents provided programmatic information and corroborated the findings of the data analysis. The Malaria Programme Reviews, conducted in collaboration with WHO by the VDCP in 2007 and 2010, were key sources for this case study [12,19].

### Programme structure

The VDCP of Bhutan coordinates and ensures the capacity of the district health teams to carry out prevention of malaria and other vector-borne diseases, namely dengue, kala-azar and Japanese encephalitis. The VDCP relies upon the structure of the national health system of Bhutan to provide the integral components of malaria surveillance, case management, and prevention through an integrated community health approach [20]. The national primary health care system is comprised of national and regional referral hospitals, district hospitals, Basic Health Units (BHUs) and Outreach Clinics (ORC). Outreach clinics conduct antenatal check-ups and immunizations, but do not play a major role in malaria control.

The service delivery structure of the VDCP is based upon multipurpose malaria health workers, termed malaria technicians, that are deployed by the VDCP to hospitals, and in the endemic southern districts, BHUs as well [12]. These health workers, whose salary is paid by the Ministry of Health, work only on malaria and provide a wide range of services including reading blood slides for malaria diagnosis, issuing treatment, case reporting, and case follow-up. They also support IRS and LLIN distribution, entomological surveillance, and Information Education and Communication (IEC) activities. Health assistants, nurses and doctors provide the malaria treatment. Village health workers help in executing IEC activities. Spray operators conduct the IRS coordinated by the malaria technicians.

The role of malaria technicians has begun to be integrated with other vector-borne diseases beyond malaria control, and a new title has been assigned--"Medical Technicians." In the future it is expected that the role of Malaria Technicians will increasingly become integrated and their main activities may change, posing a risk of diminishing vigilance for malaria as other diseases become a greater priority. However, as of yet, only a portion of them received the integration training and many are still referred to as "Malaria Technicians".

Domestic resources, through tax and non-tax revenue of the Government of Bhutan, account for two-thirds of total health-related expenditures in Bhutan, and external financing accounts for approximately one-third [21]. The Royal Government of Bhutan has provided an increasing amount of support to the VDCP over the last 5 years, contributing 21.5% of the total VDCP budget over the period 2009-2010. The total VDCP budget was $445,950 USD in 2009-2010, which included malaria and other vector-borne diseases. Government support to district malaria-related expenditures are not included in this figure.

The Government of India, a long-time partner of the Royal Government of Bhutan, has contributed USD $177,777 annually, which is mainly used to procure insecticides for Bhutan's IRS programme. This collaboration has continued since the 1960s. Approved Global Fund to Fight AIDS, Tuberculosis and Malaria (Global Fund) grant amounts were $1,737,190 (Round 4, funding received starting in 2005) and $2,662,468 (Round 7, funding began 2008) [22]. In addition, the World Health Organization (WHO) and other partners have provided technical assistance and some financial support. Out of ten countries in the South-East Asia region, Bhutan ranks the highest in cumulative per person availability of donor funding, at $10.75 USD per capita [22].

### Epidemiology of malaria

#### Locally contracted or indigenous cases

**Figure 2:**
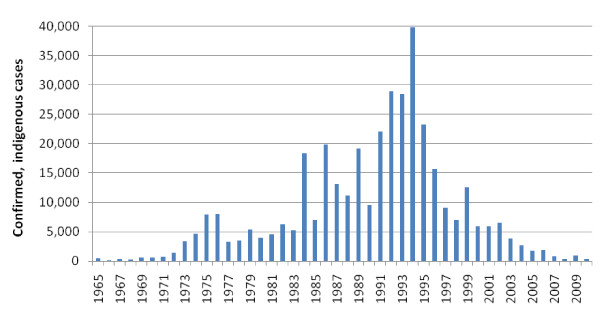
**Malaria cases in Bhutan, 1965-2010**.

In 2010, there were 436 microscopy-confirmed indigenous cases. Of these, 261 (59.9%) were due to *Plasmodium vivax*, 140 (32.1%) were due to *Plasmodium falciparum*, and 35 (8.0%) were mixed infections. The total number of cases in 2010 is similar to the 518 cases reported in 1965 (see Figure 2) [11]. All reported cases are confirmed by microscopy, even if the initial diagnosis was by RDT. Bivalent RDTs to detect both *P. falciparum *and *P. vivax *were introduced in 2006.

The highest peak of malaria cases occurred in 1994, with nearly 40,000 indigenous cases. A major decline of 85.1% occurred from 1994 (39,852 cases) to 2000 (5,935) that continued until 2010 (436 cases). A small increase in cases occurred from 2008 (329) to 2009 (972), which was associated with the early arrival of the monsoon rains or the loss of efficacy of LLINs distributed in 2006 after a lapse of 3 years [23]. The Annual Blood Examination Rate (ABER), or the number of blood slides examined for malaria parasites as a proportion of the total population at risk, varied over the years with no clear trend and ranged between 9.7% (2008) and 20.9% (2010).

The proportion of indigenous cases resulting from *P. vivax *infections, as compared to those identified as *P. falciparum *or mixed infections (both *P. falciparum *and *P. vivax*), has ranged from a low of 42.5% (2009) to a high of 59.9% (2010). Mixed infections in Bhutanese have nearly doubled from 2001 to 2010, from 4.4 to 8.0%, respectively. This increase may be related to improvement in diagnostic specificity and is less likely to reflect an increase in transmission intensity of mixed infections.

Males, specifically male farmers and students between the ages of 15-49 years, are the population groups at highest malaria risk [24]. This is most likely due to various occupational factors, such as forest work, firewood collection, guarding fields at night, or travel to India for business [8,12].

#### Imported cases

**Figure 3:**
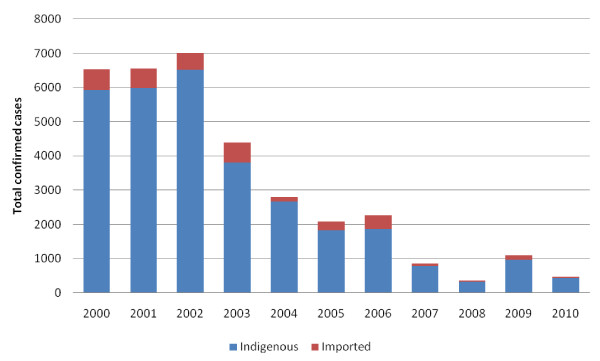
**Confirmed cases in Bhutan, 2000-2010**.

The proportion of total cases (indigenous and imported) considered imported, or those found in foreign nationals, varied greatly over the years, reaching a high of 408 imported cases in 2006, representing 21.8% of all confirmed cases (Figure 3). In 2010, 28 cases were considered to be imported (6.0% of total confirmed cases).

Imported cases have a slightly higher proportion of *P. falciparum *infections: in 2010, 46.4% of all imported infections were due to *P. falciparum *in contrast to 32.1% of indigenous cases. Similar to the increasing trend found in indigenous cases, mixed infections in imported cases appear to be increasing and represented 17.9% of infections in 2010.

**Figure 4:**
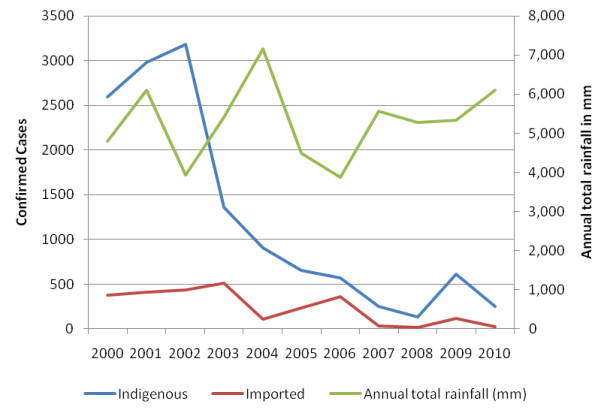
**Confirmed cases in Sarpang District, indigenous and imported cases, 2000-2010, with total annual rainfall in Sarpang District **[**25**].

Sarpang District, which borders Assam State of India, recorded the majority of imported cases (every year from 2000 to 2010) and the highest number of indigenous cases in 7 out of 10 years (2000-03, 2005, 2008-10). Over the last decade, this district has contributed an average of 87.5% of imported cases and an average of 47.1% of indigenous cases in Bhutan. The border is highly porous, exemplified by the many residents of neighboring Assam receiving treatment in the district's health clinics. In 2009 there was a reported outbreak of malaria in Assam with a 26.8% increase in reported cases [23]. This trend was mirrored in Sarpang, where a three-fold increase was reported that same year (Figure 4). Eighty percent of infections that year were due to *P. falciparum*. Rainfall trends from 2000 to 2010 from Bhur Station in Sarpang District indicate that there was no clear trend in rainfall in this district over this period (Figure 4) [25].

### Vectors

In the past, *Anopheles minimus *was incriminated in transmitting malaria in Bhutan and it was presumed that *Anopheles fluviatilis *and *Anopheles dirus *were also important vectors (See Panel 1) [26]. However, none of these species have been recorded in the last 10 years. While *Anopheles minimus *and *Anopheles fluviatilis *have been found recently in eastern Bhutan (Bhutan VDCP), *Anopheles pseudowillmori *and *Anopheles culicifacies *are suspected to be the main vectors because of their behaviour (both endo and exo-phagic and anthropophillic) and their relative abundance during the peak transmission season. Both species in high densities have been found to bite cattle between 18:00-20:00 h. Species composition studies undertaken in Bhutan on *Anopheles culicifacies *found species B and C; C is not considered a vector, but B is a proven malaria vector in India [26]. *Anopheles culicifacies *are rarely found indoors in the presence of two rounds of IRS per year and LLIN coverage. Current studies have still failed to incriminate any vectors in Bhutan. Training for sibling-species composition, host-blood meal analyses and techniques for sporozoite infectivity are needed to inform vector control interventions [12] (see Table 1).

**Table 1:** Anopheles fauna in Bhutan [26]

**Plains**:	*An. pseudowillmori, An. vagus, An. subpictus, An. culicifacies, An. jamesii, An. pseudojamesii, An. annularis, An. philippinensis*, *An. kochi, An. peditaeniatus*, *An. acinitus*, *An. barbirustris*, *An. barbumbrosus, An.umbrosus*
**Mountains**:	*An. maculatus*, *An. willmori, An. lindesayii, An. baileyii, An. aitkenii and An. bengalensis*

### Surveillance

Malaria is a notifiable disease in Bhutan, with microscopy-confirmed cases reported on a weekly basis. As private sector health practice in Bhutan is minimal, malaria under-reporting from this sector is considered negligible [2]. There is a functioning quality control system for microscopy in malaria-endemic areas; compulsory monthly blood film cross-checking is conducted by the VDCP. In at risk seasonal transmission and malaria-free areas, staff members send slides to the VDCP for cross-checking on a quarterly basis. 10% of negative and 50% of all positive slides are cross-checked for accuracy and quality. Malaria microscopy training is ordered if there are false positive or negative rates beyond the acceptable limit. A significant challenge is that blood films are sent by post and are sometimes broken and un-readable upon arrival.

Vertical reporting occurs each week wherein reports flow from health facilities to the districts, then to the VDCP [12]. Reports are submitted to the Bhutan information system of the Ministry of Health each quarter. Reporting is supervised by on-site data verification during monitoring and supervision visits to the health centers. In addition, the VDCP checks with individual centers by phone if there are missing or incomplete reports. The VDCP analyses the weekly and monthly reports and, if an increase in cases is reported, the respective health center is alerted and vector and case surveillance investigation and Information, Education and Communication (IEC) activities are carried out to locate the source of transmission and implement containment strategies, including IRS in the cases where the area has not been sprayed the two rounds that year [24]. Mass screening of fever cases is also conducted within the affected locality (no fixed radius). A team from the VDCP, accompanied by the Malaria Technician from the respective health center, conducts this investigation. Often times a report of an outbreak will reach the Health Minister.

Passive case detection (PCD) is the main method of parasitological surveillance in Bhutan. Despite the challenges of rugged terrain, health facilities with microscopy testing are available at the national, regional, and district levels. BHUs at the community level provide the bulk of malaria diagnosis using microscopy, using RDTs in the rare instances where microscopy is not available [11]. The Global Fund supported the introduction of these bivalent (*P. falciparum *and *P. vivax*) RDTs in 2006, and since that time 9,744 RDTs have been delivered to Bhutan [2]. These RDTs, if all were used (and most were used in emergencies only), represent a small portion of the total malaria tests (180,156) conducted over that time period. Back-up blood smears taken from all RDT-confirmed patients are sent to the VDCP to be tested [24]. The policy in Bhutan is to give treatment according to the RDT result in the case of RDT-testing, before confirmation of the blood smear. However, only the results of microscopy confirmation are reported.

The number of blood films collected in health facilities has declined over the past 10 years by 28.0%, with 55,046 films collected in 2010. In 2010 the ABER was 20.9%. This decline is in accord with the decline in malaria cases. Blood films collected from non-nationals varied over the last 10 years, and in 2010 there were 7,624 blood films collected from non-nationals.

Proactive case detection (ACD), or household malaria screening of those with fever by surveillance workers, was conducted in Bhutan in the 1960s and 1970s, but has not been employed since that time. The elimination strategy, beginning in 2010, calls for the reintroduction of monthly proactive ACD, or focal screening and treatment by mobile clinics, with the aim to eliminate parasite reservoirs. These clinics would be implemented by the BHUs, carried out by village health workers or volunteers, as they are located in the risk areas. Malaria screening does not occur in antenatal clinics.

### Malaria control strategies and interventions

#### Prevention and vector control

The major prevention and vector control interventions in Bhutan are IRS, ITNs, and LLINs. Larviciding and environmental management have been explored only through small-scale projects. Up until 1998, IRS was the main method of vector control, applied in malaria-endemic southern districts every 6 months with a goal of universal coverage. IRS is not conducted in other areas besides these southern districts. From 1998 to 2004, however, IRS was halted as ITN distribution became the main control tool. In 2004, IRS was re-introduced using new stratification criteria. Targeted spraying was employed according to these criteria, which were areas with confirmed malaria cases, *P. falciparum *rate above 5%, Slide Positivity Rate (SPR) above 3%, Annual Parasite Index (API) above five cases per 1,000 population, and presence of malaria deaths within the past 3 years. No IRS was used in the areas at risk for seasonal transmission in the interior. In 2006, LLINs were introduced alongside the continuation of focal IRS. From 2011 onwards, taking into account major reductions in caseload, the stratification thresholds for deployment of IRS became more stringent, to an SPR above 2% and an API above 4 per 1,000 population. Vector prevalence, vector behavior, and proximity to populations along and across the border were introduced as additional stratification criteria for use of IRS. Focal IRS is carried out in villages that do not meet the IRS stratification criteria. Focal IRS consists of spraying households within a one-kilometer radius of an indigenous case when reported. A Malaria Indicator Survey (MIS), which contained a Knowledge, Attitudes and Practices (KAP) component, was conducted in 2009 and found that 57% of respondents in the survey felt that "IRS did help in controlling the number of mosquitoes" but that many believed that "IRS effects were short-lived" [27]. 87% of households preferred using a bed net to IRS.

VDCP records indicate that population coverage of IRS, measured by the number of households sprayed out of the number of households targeted specifically for IRS, was on average 97.8% over the period 2004 to 2009. The WHO programme review similarly reports IRS coverage as "over 90%"[12]. However, population coverage estimated by the number of persons covered by IRS out of the estimated population at risk by district indicates a lower level of coverage. An average of 36.1% of the population at risk received IRS from 2004 to 2009. In 2009 coverage per person at risk peaked at 50.9%.

The Government of India has supported procurement of insecticides for the Bhutan VDCP since the 1960s. DDT (dichlorodiphenyltrichloroethane) for IRS was introduced in 1962 as part of the malaria eradication programme, and its application might have eliminated the primary vector, *An. minimus *[28]. DDT use was halted in 1995 with growing evidence of resistance in *Anopheles maculatus*. As a result, deltamethrin, a synthetic pyrethroid, was introduced for IRS and for the impregnation of mosquito nets [28]. In 2008, cyfluthrin was introduced for IRS because the VDCP could not procure adequate supplies of deltamethrin.

ITN distribution began in 1998 [29]. By the end of 2008, an estimated 90% coverage was reached in endemic areas, with two rounds annually of treatment in endemic areas and one round in epidemic areas [19]. In 2006, LLIN distribution began, with support from the Global Fund. LLINs were sent to the health centers which in turn distributed to households. While the majority of LLINs were sent to the endemic southern districts, more than 20,000 LLINs were sent to areas throughout the country considered "hard-to-reach," or more than 3 h walking distance from the nearest health center or BHU.

A total of 228,053 LLIN were distributed from 2006 to 2010. In 2010, coverage of households specifically targeted for LLIN was 96.9%, and a household survey in 2009 found that 82.5% had at least one LLIN [27]. However, when calculating 2010 coverage as the number of people protected out of the total population at risk, 77.2% of the risk population in the endemic, southern districts were protected by LLINs (assuming LLINs were appropriately used, provided protection for two people, and were effective for at least 3 years). Appropriate utilization rates of both ITNs and LLINs are estimated to be 90.1% [27].

Entomological surveillance is implemented in the endemic, southern districts and this includes vector population monitoring. Vector density studies, bio-assay tests on LLINs and susceptibility tests are conducted on a monthly basis. Insecticide resistance monitoring is conducted through three sentinel sites in Sarpang District, the border district with the highest number of cases. The elimination programme calls for the expansion of insecticide resistance studies to the areas in the interior at risk for seasonal transmission.

#### Treatment and prophylaxis

*Plasmodium vivax *infections in adults were treated with chloroquine up until 2005, when the treatment policy changed to use of primaquine (0.25 mg/kg) for 14 days and chloroquine (25 mg/kg for adults) in divided dose over 3 days. This primaquine dose is considered effective by the VDCP, although WHO guidelines suggest that in Southeast Asia higher doses are required [30]. A 2010 review of *P. vivax *treatment suggested that 0.375 mg/kg base weight is the minimum dosage to eliminate hypnozoites [31]. However, the dose has not been increased because of the risk posed to G6PD-deficient individuals. There is no point of care test for this blood disorder to use before treatment in Bhutan. Patients take this treatment at home, without observation, and are asked to report any signs indicating hemolysis. To date, there have been no reports of adverse events to the Drug Regulatory Authority regarding primaquine treatment. There are also no reports available on concerns by health workers or patients relating to use of primaquine. Treatment for uncomplicated *P. falciparum *infections from 2000 onward consisted of artesunate (3 days) with doxycycline (7 days) for adults. Artemisinin-combination therapy (ACT) was introduced in 2006 (artemether-lumefantrine). From July 2011 revised guidelines include the administration of a single dose of primaquine (0.75 mg/kg) as an anti-gametocyte for *P. falciparum *infection, without prior G6PD testing. Adult patients with mixed parasite infections receive artemether-lumefantrine (24 tablets) with primaquine (15 mg) daily for 14 days for radical cure of *P. vivax*. Malaria chemoprophylaxis is not recommended in Bhutan.

Severe and complicated *P. falciparum *infections receive artemether (3.7 mg/kg) intramuscular injection upon admission, then injections (1.6 mg/kg) once a day followed by a full course of artemisinin-combination treatment (ACT) with artemether-lumefantrine (AL) when able to tolerate oral medicines. Alternatively, intravenous administration of quinine followed by oral doses is given.

Three-day compulsory admittance to hospital is applied to all *P. falciparum *infections and patients receive directly observed therapy, with a blood slide conducted each day. Patients are then advised during discharge to return for a repeat blood slide examination after 3 days. If the patient does not return the Malaria Technician retrieves a blood slide from this person at least once. Post-treatment follow-up of *P. falciparum *cases started with Global Fund Round 4 support, but was already in practice in health centers in some endemic districts. Case follow-up of *P. falciparum *cases is now mandatory, including case investigation with monitoring of vector breeding sites conducted by BHU staff. A report form is used for this investigation, capturing information on patient travel history, reported adherence to treatment, household residents, LLIN condition, IRS coverage, and potential breeding sites. Twenty-eight day follow-up of *P. vivax *infections to measure treatment adherence and efficacy is planned but not yet introduced.

Since 1984 drug efficacy monitoring for the most part has focused on treatment of *P. falciparum*. In 2006, five sentinel sites were established in endemic districts to monitor drug resistance to ACT, and the efforts were further boosted by Global Fund support (Round 7). The ACT AL has been shown to be 100% efficacious for the treatment of *P. falciparum*, according to the VDCP. The elimination strategic plan calls for therapeutic efficacy studies of *P. vivax *treatment.

### Enabling and challenging conditions in malaria control and elimination

In addition to the national programme strategies and interventions developed and implemented to control and eliminate malaria, there are also environmental and socio-economic factors that can directly impact malaria transmission. These factors are considered below.

#### Enabling conditions

Bhutan has made major advances in economic development in the last decade. Gross National Income (GNI) per capita nearly tripled from 2000 and 2009, from $730 to $2,030 (current USD, Atlas Method), the latter figure being the highest GNI per capita in South Asia [21]. Road length increased by 43% from 2001 (3,746 km) to 2008 (5,363 km). Tourism revenue more than quadrupled during the same period ($9.2 million USD to 38.8 million).

In addition to economic advances, Bhutan has strengthened its health system and offers free health services for all. The WHO awarded the country its 50^th ^anniversary award for primary health care in 1998, referring to its system as "one of the best in South-East Asia" [32]. The country's elimination agenda benefits from a stronger health system than exists in most lower-middle income countries. Bhutan is currently one of the leading countries in per capita expenditure on health, on par with Sri Lanka, spending up to $75 per capita (current USD) [21].

From 2000 to 2009, there was a 61% increase (109-176) in the number of physicians in the national health system [33]. There was also an increase in births attended by trained personnel, from 24% in 2005 to 66.3% in 2008 [34]. The malaria programme also benefits from a strong national supply and logistics system: for example, there were no reported anti-malarial drug stock-outs in recent years [12]. District and sub-district health facilities coordinate movement of supplies to avoid stock-outs.

Bhutan's health services are nearly all provided by the public sector. There are no private medical facilities and only a handful of retail pharmacy shops [12,35]. As a result, the government has a high level of control of case management and malaria control measures.

#### Challenging factors

Rugged terrain and remote, hard-to-access population groups create challenges in access to healthcare facilities. The majority of the population (69.1%) live in rural areas [5]. As stated above, 21% of the population are considered "difficult to access" in that they are located more than 4 h walk from the nearest road. Access is further impeded by rain, landslides and road closures during the major monsoon season, which is also the peak season of malaria transmission. Delays in treatment have been associated with remoteness and the cost of transportation to health facilities [35]. In addition, there is a strong tradition of traditional medicine in Bhutan whose practice can delay prompt and correct malaria treatment [35].

Although, over the last decade there have been recent improvements in the number of staff available in the national health system, historically there have been shortages of highly trained workers as a result of the limited training institutions located in Bhutan. There is no medical college and physicians and most technical professions are trained abroad [8,21].

While the socio-economic development taking place in Bhutan may play a role in reducing the receptivity to malaria transmission, there are major development projects underway that could undermine those advances. Major construction of hydropower dams and other projects may expand vector-breeding habitats and have led to large influxes of migrant workers, typically from malaria endemic regions of India and Bangladesh, thereby increasing the risk of importation of malaria and onward transmission. There are an estimated 35,000 documented migrant workers in Bhutan, the majority of which are employed in large-scale development projects in the interior and southern districts. While a recent cross sectional survey conducted in two hydroelectric plant construction sites indicated a low level of parasitaemia in worker groups, the overall risk posed by these migrants is not known [36]. The majority of workers surveyed were from the Indian states of West Bengal, Bihar, Jharkhand, and Uttar Pradesh [36]. Outside of migrant labor, further population movement results from the national resettlement programme, which relocates Bhutanese from low transmission areas to endemic areas to increase access to arable land. These resettled populations may both lack malaria immunity and knowledge about the disease and its prevention and treatment [35].

Thirdly, there have been recent short and long-term changes in climate and rainfall, which are thought to have contributed to the increase in incidence in 2009 [12]. Climate-related severe weather patterns have been observed, and increasing temperatures and changes in rainfall are predicted to have major health impacts [37].

Lastly, and perhaps the greatest threat to a successful elimination plan, is the border with Assam State of India. The porous border is malaria-endemic, largely composed of forest reserve, and is characterized by historical political instability, transient and semi-permanent settlements, mobile populations, and impoverished conditions. There are virtually no malaria surveillance or referral services, apart from non-governmental organizations (NGOs) based in the area [23]. As a result, Assam population groups often migrate into Bhutan seeking healthcare services, in particular at Sarpang District Hospital. Other migrant groups include daily contractual workers and casual laborers. There is an estimated daily migration of 1,000 people entering Bhutan through each regulated checkpoint in ten border towns. It is unknown how many migrants pass through unregulated areas of the border, and as these populations have not been studied, there are no certain estimates of cross border movement. A clearer understanding of the migration pathways into and within Bhutan would help in targeting interventions to prevent importation of malaria.

### Elimination strategy

The decision to pursue malaria elimination in Bhutan, which aims to eliminate first in the interior of the country and progressively work toward the southern border areas, was shaped by sustained low malaria incidence in the interior of the country over the last 10 years. In most of this area there have been no indigenous cases of malaria in 3 years, with few imported from the border districts. In addition, the epidemiological, technical and programmatic assessment of the malaria programme review, conducted in March 2010 in collaboration with WHO, supported the decision to pursue elimination [12]. Progress made in the South-East Asia region, such as recent successes in Sri Lanka and Thailand, also influenced the decision to eliminate [38].

Progressive malaria elimination in Bhutan will require intensified efforts in case-based surveillance, with rapid notification, case investigation and case containment strategies. The expansion of parasitological and entomological surveillance is a priority, and must include the identification and mapping of local malaria foci, which is dependent upon the creation of district-level case investigation and rapid response teams. The expansion of outreach clinics, typically used for vaccination and antenatal activities, to include malaria PCD and methods of ACD will enhance surveillance in remote areas. Case management policies will also be strengthened--case follow-up for one month for *P. falciparum *infections is planned, and a 14-day follow-up period for *P. vivax*. Case-based IRS, when indigenous cases are found, is planned to be implemented in the interior districts, where IRS has not yet been employed.

A cross-border malaria strategy with India has been identified as a necessary next step in order to achieve elimination in Bhutan. Over the years, several efforts were made to establish cross-border mechanisms for Bhutan and India to improve malaria control, surveillance, information sharing and research along the border zone. In the early years of the malaria programme, in the 1960s, IRS activities were synchronized along the border. In the mid-1990s, WHO supported meetings between the countries seeking to improve information-sharing through study tours, conduct joint training and strengthen entomological surveillance. However, these activities were not sustained.

In 2000, the USAID Bureau for Asia and the Near East (ANE) and USAID Nepal, in collaboration with WHO, supported a regional initiative of Bangladesh, Bhutan, India, and Nepal (BBIN) to implement cross-border activities for control of malaria, leishmaniasis, and Japanese encephalitis. The goal was to support the development of new interventions, expansion of proven interventions, and to improve surveillance programmes. Guidelines for surveillance, research studies, an IEC national programme, and a surveillance system were developed for Bhutan. The BBIN project was eventually disbanded due to a reduction in funding.

The current elimination strategy focuses mainly on management of imported malaria. Six border malaria screening centers will be installed at security checkpoints in five districts. The planned border malaria screening and LLIN distribution will target both at-risk Bhutanese and migrant workers in construction sites.

## Discussion

Bhutan has achieved a 98.7% decrease in microscopy-confirmed malaria cases from 1994 to 2010. Declines occurred in the zone at risk for seasonal transmission in the interior of the country as well as in the endemic, southern border districts [24]. In 2004, Bhutan met and surpassed the Millennium Development Goals set by RBM, achieving over 50% reduction in cases well ahead of the 2010 goal. Stemming from this success, and building on the strengths of the national health system and the Vector-borne Disease Control Programme, Bhutan is embarking on malaria elimination.

The evidence-based strategies implemented by the VDCP are likely the root of Bhutan's malaria success, along with the economic and social development seen in the country. The programme benefits from a strong primary health care system and continually expanding access to health care, including malaria diagnosis and treatment, at the district and sub-district levels in rural and remote areas. A well-functioning health supply system allows few stock-outs. As a result of these improvements, access to timely diagnosis and treatment through PCD has likely improved, with weekly case reporting linking epidemiological trends to vector control measures. Evidence-based case management policies, including the implementation of ACT for *P. falciparum *cases, may have also contributed to the declining transmission [24].

The deployment of IRS, ITN, and LLIN with the use of strong stratification criteria has resulted in high coverage of targeted populations most at-risk, contributing to the downward trend in incidence [6,24]. Global Fund grant support increased access to these prevention measures.

In order to maintain the progress of the last decade, Bhutan must address the challenges it faces to eliminate malaria. The increase in cases that occurred from 2008 to 2009 is an indicator that there is still more work to be done. Further studies on understanding mosquito vectors and their bionomics are warranted in order to formulate more specific intervention strategies. The Malaria Technicians deployed by the VDCP are a pillar of the programme and must be maintained in order to ensure vigilance and timely response. The integration of duties of Malaria Technicians could potentially lead to a weakened response to malaria outbreaks and this must be avoided. Increases in transmission across the southern border in Assam or West Bengal, India may directly impact transmission in southern Bhutan [24]. Adding to the risk is the continual migration into Bhutan from these states, regulated and unregulated and daily and long-term, which may continually reintroduce infections into all receptive areas of the country [7]. A clear understanding of the origin and pathways of migrants into Bhutan would facilitate the development of effective strategies to mitigate and manage imported malaria and the risk of onward transmission.

### Limitations

This case study is based on a retrospective analysis of national surveillance data on confirmed malaria infections. The number of unconfirmed cases is not known, yet the relatively high level of access to public health facilities and lack of private sector facilities translates to a negligible level of unconfirmed infections. The epidemiology data does not allow for a more extensive analysis of the malaria infection of long-term migrant workers in Bhutan. National case investigation procedures have not collected enough information to identify the origin of infection, but will attempt to do so in the future.

## Conclusions

Bhutan has made great strides towards elimination. The greatest challenge to this goal is in identifying and containing imported infections from the neighbouring Indian states. The malaria programme has identified two main approaches to face this problem. Firstly, implement border screening and secondly, develop cross-border and regional malaria collaborations [7]. A recent WHO report recommends border post screening for malaria not only to identify and treat infections, but also to install a way to measure increases in transmission in order to adequately prepare response measures [36]. Overall, though, evidence is lacking on the impact of border screening, with only a few available examples, most from island contexts which are obviously very different than landlocked Bhutan. The Thailand-Cambodia artemisinin resistance containment project has included mobile malaria clinics at border crossings in Thailand, but this activity has not been assessed for impact on transmission reduction [39]. Recent research on a passenger screening surveillance programme in Mauritius [40] and the acceptability of inter-province port screening in Solomon Islands [41] provides some examples of identification of imported malaria infections, but more research is needed. In Bhutan, where borders are porous and migrant populations pass daily over the border, other measures may be needed in addition to the border screening centers, which target longer-term migrant workers. Regional or cross-border initiatives may be an important tool to lower importation risk [1,7]. Harmonized surveillance, case management and vector control strategies and their synchronized implementation in border regions are generally successful through a multi-country platform. Yet the history of cross-border collaboration between Bhutan and India attests to the challenge of developing such an initiative, from getting the key partners to the table to finding sustainable funding support. The cancellation of Rounds 11 and 12 by the Global Fund speaks to the latter challenge. As more countries near elimination, regional approaches, backed by sound evidence and supported with adequate funding, are likely to be the way forward.

## Competing interests

TY and RN are supported by the Vector-Borne Disease Control Programme, Ministry of Health, Bhutan. TY represents Bhutan in the Asia Pacific Malaria Elimination Network (APMEN). RG is Lead of the Malaria Elimination Initiative at the UCSF Global Health Group, where CSG is Program Coordinator. Both RG and CSG provide assistance to the APMEN Joint-Secretariat. The Global Health Group provides support to eliminating countries, such as Bhutan, and is funded by the Bill & Melinda Gates Foundation and ExxonMobil. APMEN is funded through a grant from the Australian Agency for International Development (AusAID). VD is supported by the National Institute of Malaria Research (ICMR) of India and has served as a WHO consultant in Bhutan. GNLG is supported by the Anti-Malaria Campaign, Ministry of Health, Sri Lanka and also has served as a WHO consultant in Bhutan. GNLG is also a representative of Sri Lanka in APMEN. KT is supported by the Malaria Unit, South-East Asia Regional Office of the World Health Organization and has a non-voting position on the APMEN Advisory Board. SM is supported by the World Health Organization Global Malaria Programme.

## Authors' contributions

All authors contributed by guiding and shaping the messages and ideas contained in this paper. The text was drafted by TY, CSG, RG, and VD. TY provided in-country guidance during data collection. TY and CSG collected the programme data. CSG conducted the data analysis. All authors took part in the review, preparation, and final approval of the manuscript.

## Supplementary Material

Additional file 1 **Webappendix**. A Literature Review on Malaria Control and Elimination in Bhutan. Click here for file
