# Supplementary material for: Malaria control in Bhutan: case study of a country embarking on elimination
Source: Malar J. 2012 Jan 9;11:9. doi: 10.1186/1475-2875-11-9 (PMC3278342; doi:10.1186/1475-2875-11-9)
Supplement: Additional file 1 — Webappendix. A Literature Review on Malaria Control and Elimination in Bhutan. [file 1475-2875-11-9-S1.DOCX]

**Webappendix: A Literature Review on Malaria Control and Elimination in Bhutan**

The literature review identified the following publications, WHO reports and documents, reports and documents from the Bhutan Ministry of Health and other ministries, related other reports, and news articles.

**Peer-review publications on Bhutan**

Andersen F et al: **Trends in malaria research in 11 Asian Pacific countries: an analysis of peer-reviewed publications over two decades***.* *Malaria Journal* 2011, **10:**131.

Baral LR: **Bilateralism under the Shadow: the Problems of Refugees in Nepal-Bhutan Relations***.* *Nepalese Studies* 1993, **20**.

Belfiglio VJ: **India's Economic and Political Relations with Bhutan***.* *Asian Survey* 1972, **12:**676-685.[ http://www.jstor.org/stable/2643108]

Dev V: **Anopheles minimus: its bionomics and role in the transmission of malaria in Assam, India***.* *WHO Bulletin OMS* 1996, **74:61-66**.

Dev V, Dash AP, Khound K: **High-risk areas of malaria and prioritizing interventions in Assam.** *Current Science* 2006, **90:**32-36.

Dev V, Phookan S, Sharma VP, Anand SP: **Physiographic and entomologic risk factors of malaria in Assam, India.** *The American Society of Tropical Medicine and Hygiene* 2004, **71:**451-456.

Dua VK, Dev V, Phookan S, Gupta NC, Sharma VP, Subbarao SK: **Multi-drug resistant Plasmodium falciparum malaria in Assam, India: Timing of recurrence and anti-malarial drug concentrations in whole blood.** *The American Society of Tropical Medicine and Hygiene* 2003, **69:**555-557.

Chatterjea JB: **Haemoglobainopathies, Glucose-6-phosphate Dehydrogenase Deficiency and Allied Problems in the Indian Subcontinent***.* *Bulletin of the World Health Organization* 1966, **35**:837-856.

Crossette B: **Bhutan: When Environment Drives Public Health Policies***.* *Disease Control Priorities Project* 2007 [<http://www.dcp2.org/features/45>].

Glover SW, Dema R, Yangzom P, Sonam K, Gleghorn C.: **A review of health and access to health information in Bhutan***.* *Health Information and Libraries Journal* 2006, **23**:283–297.

Hutt, M: **Ethnic Nationalism, Refugees and Bhutan***.* *Journal of Refugee Studies* 1996, **9.**

The Journal of the American Medical Association: **International Notes: Surveillance of Health Status of Bhutanese Refugees-Nepal, 1992***.* *JAMA: The Journal of the American Medical Association* 1993, **269**:846-850 [ http://jama.ama-assn.org/content/269/7/846.short].

Kondrashin A: **Malaria in the WHO Southeast Asia Region***.* *Indian J Malariol* 1992, **29**:129-60.

Kumar A, Valecha N, Jain T, Dash AP: **Burden of Malaria in India: Retrospective and Prospective View***.* *The American Society of Tropical Medicine and Hygiene* 2007, **77**:69-78.

Malaria Atlas Project (MAP): The Spatial Distribution of Plasmodium falciparum malaria endemicity in Bhutan [http://www.map.ox.ac.uk/media/maps/pdf/mean/BTN_mean.pdf].

Narain JP: **Malaria in the South-East Asia Region: Myth and Reality***.* *Indian J Med Res* 2008, **128**:1-3.

Proctor MH: **Human Rights Violations Among Bhutanese Refugees***.* *JAMA: The Journal of the American Medical Association* 1995, **274:**1891-1892.

Rajagopal R: **Studies on Malaria in Bhutan***.* *Journal of Communicable Diseases* 1985, **17**:278-286.

Rudra SK, Mukhopadhyay A: **Mosquito Species Composition of the Dooars of West Bengal, India.** *The Zoological Society* 2010, **63**:21-25.

Sharma PK et al: **A malaria outbreak in Naxalbari, Darjeeling district, West Bengal, India, 2005: weaknesses in disease control, important risk factors***.* *Malaria Journal* 2009, **8**.

Shaw BC: **Bhutan in 1992: "Going Out" and "Staying Back***.***"** *Asian Survey* 1993, **33**:141-144 [http://www.jstor.org/stable/2645322].

Tobgay T, Lhazeen K: **Malaria perceptions and practices in Bhutan***.* *Southeast Asian J Trop Med* *Public Health* 2010, **41:**274-9.

Tobgay T, Torres CE, Na-Bangchang K: Malaria prevention and control in Bhutan: Successes and challenges [http://www.sciencedirect.com/science/article/B6T1R-51JXFNG-2/2/5e821d3916006c6b155d0966910310e0].

Wadhwa VAD: **Malaria in South Asia: Lessons from the Past, View to the Future: Summary and Concluding Remarks.** *Advances in Asian Human-Environmental Research* 2010, 179-200.

Wangchuk P et al: **Antiplasmodial activity of atisinium chloride from the Bhutanese medicinal plant, Aconitum orochryseum***.* *Journal of Ethnopharmacology* 2010, **130**:559-562.

Wangdi K et al: **Development of temporal modelling for forecasting and prediction of malaria infections using time-series and ARIMAX analyses: A case study in endemic districts of Bhutan.** *Malaria Journal* 2011, **9**:251.

Wangdi K, Kaewkungwal J, Singhasivanon P, Silawan T, Lawpoolsri S, White NJ: **Spatio-temoral patterns of malaria infection in Bhutan: a country embarking on malaria elimination***.* *Malaria Journal* 2011, 10:89.

Wangchuk P et al: **Antiplasmodial agents from the Bhutanese medicinal plant Corydalis calliantha***.* *Phytotherapy Research* 2010, **24**:481-485.

Ward M, Jackson F: **Medicine in Bhutan***.* *The Lancet* 1965, **285**:811-813.

**Publications related to malaria elimination**

Feachem RGA et al: **Shrinking the malaria map: progress and prospects***.* *The Lancet* 2010, **376:**1566-1578.

Lee PW, Liu CT, Rampao HS, do Rosario VE, Shaio MF: **Pre-elimination of malaria on the island of Principe***.* *Malaria Journal* 2010, **9:**26.

Moonen B et al: **Operational strategies to achieve and maintain malaria elimination***.* *The Lancet* 2010, **376**:1592-1603.

Noor A et al: **Establishing the extent of malaria transmission and challenges facing pre-elimination in the Republic of Djibouti***.* *BMC Infectious Diseases* 2011, **11:**121 [http://www.biomedcentral.com/1471-2334/11/121].

O'Sullivan M et al: **Malaria elimination in Isabel Province, Solomon Islands: establishing a surveillance-response system to prevent introduction and reintroduction of malaria***.* *Malaria Journal* 2011, **10:**235.

Tatarsky A et al: **Preventing the Reintroduction of Malaria in Mauritius: A Programmatic and Financial Assessment***.* *PLoS ONE* 2011, **6**:e23832.

**WHO reports and documents**

Dev V: **Strengthening the capacity of Bhutan malaria control programme in response to epidemics with particular emphasis on vector surveillance and cross-border activities***.* World Health Organization, Assignment Report TRL53651 2010.

World Health Organization. **Achievement of universal immunization in the Kingdom of Bhutan: a joint Government/UNICEF/WHO Epi survey and review, 25 January to 13 February 1991.** WHO Project: ICP EPI 001 SEA/EPI/98 1991: p. 1-32.

World Health Organization. **Alternative Approaches to Vector Control, Report of an Intercountry Consultative Meeting, Yangon, Myanmar, 4-7 May 1999***.* WHO Project: ICP CTD 001 SEA-VBC-71 2000.

World Health Organization: **Bhutan Malaria Control Programme, Report of the Review 12-16 February 2007.** World Health Organization, Regional Office for South-East Asia, SEA-MAL-248 2007: p. 1-37.

World Health Organization: **Bhutan Malaria Control Programme Review : A Report**. World Health Organization 23-30 March 2010 [<http://203.90.70.117/PDS_DOCS/B4669.pdf>]

World Health Organization. **Cross-border Control of AIDS, TB and Malaria in Pilot Districts of Bhutan and India, A joint plan of action, 2002***.* WHO Project: ICP OSD 001 2002: p. 1-23.

World Health Organization: **Cross-border initiatives on HIV/AIDS, TB, malaria and kala-azar: report of an intercountry meeting, Kathmandu, 6-9 March 2001***.* WHO Project: ICP OCD 041 SEA-CD-123 2001: p. 1-20.

World Health Organization: **Cross-border initiatives on priority communicable diseases: report of an intercountry meeting, New Delhi, India, 24-27 July 2001***.* WHO Project: ICP CPC 003 2002: p. 1-16.

World Health Organization: **Development of South-Asia Surveillance Network for Malaria Drug Resistance, Report of an Informal Consultative Meeting, New Delhi, India, 9-10 January 2002***.* WHO Project No: ICP CPC 400 2002: p. 1-14.

World Health Organization: **Malaria Profile of SEA Region***.* 2009.

World Health Organization. **Managing the outbreak of malaria, to be used by the Border Districts of Bangladesh, Bhutan, India, Myanmar, Nepal, and Thailand.** WHO SEA/MAL/195 1997: p. 1-23.

World Health Organization: **Operational Guidelines on Cross-Border Control of Priority Communicable Diseases***.* WHO Project: ICP CPC 001 2001: p. 1-12.

World Health Organization: **Population at Risk of Malaria in SEA Region***.* 2009.

World Health Organization: **Quality assurance and accreditation: report of an intercountry consultation, Yangon, Myanmar, 16-19 November 1999***.* WHO Project NO: ICP THC 001 SEA-HLM-323 2000: p. 1-13.

World Health Organization. **SEA Region Revised Malaria Control Strategy 2008**. New Delhi: Regional Office for South-East Asia.

World Health Organization: **World Health Organization Library Information System** [http://apps.who.int/whosis/en/].

World Health Organization Bhutan Country Office: **WHO Bhutan Health Information***.* WHO Country Office for Bhutan, 2011.

World Health Organization Bhutan Country Office: **Health Information: Country Health Profiles***.* WHO Country Office for Bhutan, 2011.

World Health Organization Global Malaria Programme: **Global Plan for Artemisinin Resistance Containment (GPARC)***.* World Health Organization, 2011: p. 1-87.

World Health Organization Global Malaria Programme: **Malaria Elimination: A field manual for low and moderate endemic countries,** 2007: p. 1-85.

World Health Organization Global Malaria Programme: **World Malaria Report, 2010.** 2010: p. 1-204.

World Health Organization SEARO: **Bhutan Malaria Control Programme Review: A Report, 2010** [http://203.90.70.117/PDS_DOCS/B4669.pdf].

World Health Organization SEARO. **Bhutan Malaria Control Programme. Report of the Review 12-16 February 2007**. New Delhi: Regional Office for South-East Asia, World Health Organization. SEA-MAL-248 2007.

World Health Organization SEARO: **Country Health System Profile***.* WHO SEARO, 2011.

World Health Organization SEARO. **Development of Strategies and Approaches to Malaria Control in South-East Asia, Report of a Regional Technical Consultation, 1987**. New Delhi: World Health Organization SEARO 1987.

World Health Organization SEARO: **Malaria Profile - Bhutan***.* WHO SEARO, 2010.

World Health Organization SEARO: **Malaria Situation in SEAR Countries - Bhutan***.* 2011.

World Health Organization SEARO Malaria Unit: **Malaria Control in South-East Asia Region Presentation** 2010.

**Bhutan Ministry documents (from Ministry of Health, Ministry of Economic Affairs, other)**

Gross National Happiness Commission, Ministry of Health Bhutan: **Annual Report, Strengthening Malaria Prevention and Control in Bhutan, Global Fund to Fight HIV, Tuberculosis, and Malaria.** 2008: p. 1-18.

Ministry of Economic Affairs, Meteorology Unit: **Bhur Meteorological Data, 2008 to 2010***.* 2011.

Ministry of Health, Bhutan:

**Annual Health Bulletin, 2010***.* p. 1-114.

**Final Report, Health Sector Review, Bhutan, 10-27 January 2007***.* p. 1-76.

**Operational Guideline for Community Action Group Formation at Chiwog Level and Training Module for CAG Members***.* 2009.

Ministry of Labour, Bhutan:

**Handbook on Recruitment and Employment of Foreign Workers in Bhutan***.*

**Labour and Employment Act, 2007***.* Royal Government of Bhutan.

**Labour Market Information Bulletin, 2009.**

National Environment Commission: **Strategizing Climate Change for Bhutan***,* 2009. Royal Government of Bhutan.

National Statistical Bureau:

**Bhutan 2010 Data Sheet***.* Royal Government of Bhutan.

**Bhutan at a Glance. 2005 - 2010**. Royal Government of Bhutan.

**Population Projections of Bhutan 2005-2030***.* Royal Government of Bhutan, 2005.

**Results of Population & Housing Census of Bhutan 2005 and 2006.**

**Socio-economic and Demographic Indicators, 2005 and 2008.**

**Statistical Yearbook of Bhutan (December 2005, November 2006, October 2009, November 2010)***.* Royal Government of Bhutan 2005.

Tschering D, Sithey G: **Climate Change and Health in Bhutan.** Capacity Strengthening in the Least Developed Countries (LDCs) for Adaptation to Climate Change (CLACC). CLACC Working Paper 4, 2008.

Vector-borne Disease Control Programme:

**Annual Malaria Report 2007 (DRAFT)**. 2007. Department of Public Health.

**Bhutan National Malaria Control Strategy, 2008-2013***.* 2008.

**Malaria Indicator and Bed Net KAP Study 2009, Bhutan***.* p. 1-80.

Zangpo K, Z.N., Poulsen K., *A Study on Knowledge, Attitude and Practice about Malaria Awareness and Bed Net Use.* 2002.

**Other reports**

Asian Development Bank: **Bhutan: Health Care Reform Program***.* ADB Completion Report, 2006. Project Number: 33071, Loan Number: 1762(SF): p. 1-49.

Bhutanese Community Profile. Commonwealth of Australia, June 2007 [<http://www.immi.gov.au/living-in-australia/delivering-assistance/government-programs/settlement-planning/_pdf/community-profile-bhutan.pdf>].

Brantly E, Wijeyaratne P, Singh D, Pandey S: **Intercountry collaboration for improving surveillance and control of vector-borne diseases***.* EHP: Activity report 136 2004: p. 1-38.

Crossette B: **Bhutan: When Environment Drives Public Health Policies***.* Disease Control Priorities Project 2007: p. 1.

Dorji C: **Bhutanese Health Care Reform: A Paradigm Shift in Health Care to Increase Gross National Happiness.** p. 413-436.

Mittal PK, Wijeyratne P, Pandey S: **Activity Report 129: Status of Insecticide Resistance of Malaria, Kala-azar and Japanese Encephalitis Vectors in Bangladesh, Bhutan, India and Nepal (BBIN)***.* Environmental Health Project, Activity Report 129 2004: p. 1-98.

National Institute of Malaria Research: **Annual Report 2008-09**. Indian Council of Medical Research. pp. 1-85.

National Institute of Malaria Research: **Estimation of the True Malaria Burden in India: A Profile of the National Institute of Malaria Research**. p. 91-99

Wijeyaratne DPM: **Presentation: EHP Experience in Malaria at a Cross-border Inter-country Level in Bangladesh, Bhutan, India and Nepal (BBIN).** 2003.

Wijeyaratne PM, Valecha N, Joshi AB, Singh D, Pandey S: **An inventory on malaria drug resistance in Bangladesh, Bhutan, India and Nepal.** Environmental Health Project, Activity Report 130, 2004: p. 1-43.

**News articles**

Bhutan Today**: Combating HIV, TB and Malaria***.* *Bhutan Today*, 2010. Wednesday, February 23rd, 2011.

Zam D: **Health Journey: Laudable but challenging***.* *Bhutan Today*, 2010: p. 1.
